# Anti-*Cryptosporidium* oocysts polyclonal antibodies for cryptosporidiosis diagnosis and protection

**DOI:** 10.1186/s13568-023-01632-w

**Published:** 2023-11-09

**Authors:** Alyaa Farid, Silvia Aiad, Gehan Safwat

**Affiliations:** 1 Biotechnology Department, Faculty of Science, Cairo University, Giza, Egypt; 2 Faculty of Biotechnology, October University for Modern Sciences and Arts (MSA), Giza, Egypt

**Keywords:** *Cryptosporidium parvum*, Polyclonal antibodies, Oocysts, Immunoglobulin A, Interferon-γ, Interleukin-4

## Abstract

Cryptosporidiosis is an intestinal infection that is triggered by the protozoan parasite *Cryptosporidium* spp. *Cryptosporidium* oocysts can spread from one host to another either through direct contact with infected hosts' faeces or through indirect means (consumption of contaminated water or food). Significant numbers of oocysts are produced as a result of the rapid growth of the parasite within the infected hosts. For proper care of cryptosporidiosis, a laboratory diagnosis is necessary. Therefore, this study aimed to produce anti-*Cryptosporidium parvum* (*C. parvum*) oocyst immunoglobulin (Ig)G polyclonal antibodies (pAbs). The produced pAbs were used in the detection of *C. parvum* oocysts antigens in stool and serum samples of infected calves. Moreover, pAbs were tested in protection of balb-c male mice from cryptosporidiosis infection. *C. parvum* oocysts were used in the preparation of antigens to be used in the immunization of New Zealand white rabbits. pAb was purified by ammonium sulphate precipitation method, caprylic acid purification method and diethylaminoethyl (DEAE) anion exchange chromatographic method. Sandwich enzyme-linked immunosorbent assay (ELISA) (using prepared pAb) scored higher sensitivity (85% and 95% for serum and stool samples) than that (80%) of microscopic examination of stool samples. Moreover, pAb significantly reduced the oocysts shedding, decreased inflammatory cytokines and enhanced the loss in the body weight of protected animals. The prepared pAb succeeded in the diagnosis of cryptosporidiosis in calves with high sensitivity either in the serum or stool samples. Our results indicated the usefulness of using the prepared pAb in protection against cryptosporidiosis.

## Key points

1. Importance of pAb in the diagnosis of cryptosporidiosis in calves.
2. Protection of balb-c mice by anti-*Cryptosporidium* oocysts pAb.
3. Sandwich ELISA has a higher sensitivity than microscopy.

## Introduction

Livestock offers financial possibilities, draught power, revenue to rural communities, and an option of foreign finances for the country (Ebiyo and Haile 2022). The capacity to reproduce and the survival of calf are the two factors that mostly affect the cattle productivity. Worldwide, dairy farmers face serious issues with calf mortality and morbidity (Gebremedhin 2014). The majority of calves’ deaths are caused by infectious parasites. The most typical disease symptom in newborn calves is diarrhea, which is responsible for roughly 74% of the calves’ mortality in the first two weeks after birth (Birhan et al. 2019). *Cryptosporidium (C.)*, Rotavirus, *Salmonella*, *Escherichia coli* and *Giardia* are some of the most frequent causes of diarrhea in calves (Mullusew et al. 2020).

Calves are susceptible to the zoonotic disease cryptosporidiosis, which is brought on by a parasitic protozoan of the species *C.* According to Shafieyan et al. (2014), *C.* can grow and replicate in the intestinal tracts, causing diarrhea in the infected host. According to Wegayehu et al. (2013), this condition has a significant economic effect due to its poor performance, expensive treatment costs, high mortality and morbidity. According to Manyazewal et al. (2018), cryptosporidiosis is a serious public health problem. Cattle are thought to be significant potential reservoirs for human infection. According to Gharpure et al. (2019), infections are often spread by eating, inhalation, and collateral contact with infectious stages of fully sporulated oocyst. There are over forty different genotypes of *Cryptosporidium* and about twenty four different species, with *C. parvum*, *C. andersoni*, *C. ryanae*, and *C. bovis* being the most prevalent. Cryptosporidiosis affects a wide variety of hosts and is present throughout the world (Vohra et al. 2012). *C.* was initially discovered in immunocompromised Arabian horse foals in 1978 (Snyder et al. 1978). At first, none gave the parasite any attention. Cryptosporidiosis became well-known owing to the historical events behind the deaths of patients with AIDS who had contracted *C.* in the late nineteenth century (Li et al. 2022). Due to *C.'s* widespread distribution, infections with the organism have been reported in more than 70 different countries (Thompson and Ash 2016).

The identification of protozoan parasite can be done directly using parasitological methods or indirectly using serum circulatory antigens and antibody detection (Farid 2023). One of the most often employed methods, for cryptosporidiosis diagnosis, is the microscopical evaluation. In this method, the parasite's oocysts in stool samples are commonly examined using direct optical microscopy for the laboratory diagnosis of cryptosporidiosis. An experienced examiner is required to detect the presence of *Cryptosporidium* and the phase of its life cycle (McHardy et al. 2014). However, this method has a number of shortcomings, including the lengthy time frame needed, significant oocysts variability, and low infection rates (Van Dam et al. 2004). Contrarily, immunodiagnostic methodologies offer much higher sensitivity and usability in addition to a variety of antibody testing techniques that have been created for the early identification of protozoan parasite (Maher et al. 2022). Immunological techniques can be used to diagnose protozoa by detecting antibodies [immunoglobulins (Ig) G or IgM] or antigens using enzyme-linked immunosorbent assay (ELISA), immunofluorescence assays, and/or western blots (Júlio et al. 2012). Although, IgM appeared to be a successful detector in severe infections, it has cross-reactivity with other protozoans. Moreover, IgG remains in the serum for 18 months while IgM amount decreases after 2 to 3 weeks of infection (Pacheco et al. 2020). As a result, antibody screening tests run the risk of returning a false positive result. With a specificity of 92.72% and a sensitivity of 97%, the sandwich ELISA approach with polyclonal antibodies (pAbs) has been successful in detecting several parasite types in feces samples (Maher et al. 2022). Therefore, antigen screening tests is more recommended due to the high sensitivity that it can offer, in addition to, its reliability in acute infection (Khurana and Chaudhary 2018).

Over a hundred medications have been investigated in the treatment of cryptosporidiosis, but none have yet been authorised for use in the prevention or treatment. Several studies used different approaches to protect and/or prevent calves from cryptosporidiosis infection. These results of Jenkins et al. (2004) suggested that inoculating calves with *C. parvum* oocysts exposed to gamma radiation (400-Gy) may be able to protect them against cryptosporidiosis. Focusing on the anti-P23 yolk antibody in animals and immunocompromised individuals, it would be able to develop a passive immunisation method against *C. parvum* (Omidian et al. 2014). In order to avoid *C. parvum* infection, Lefkaditis et al. (2020) demonstrated the significance of IgG levels in the colostrum given to newborn calves on their first day of life. However, given that numerous studies have shown the effectiveness of passive immunotherapy by hyperimmune bovine colostrum in decreasing the oocysts shedding and symptoms in humans, lambs, and calves (Fayer et al. 1989; Naciri et al. 1994; Perryman et al. 1999). Only two other investigations employed the pre-parturient sheep in the production of hyperimmune anti-*C.* colostrum (Naciri et al. 1994; Jenkins et al. 2004).

Therefore, the study aimed to produce anti-*C. parvum* oocyst IgG pAb. The produced pAb was used in the detection of *C.* oocysts antigens in the stool and serum samples of infected calves. Moreover, pAbs were tested in the protection of balb-c male mice from cryptosporidiosis infection.

## Materials and methods

### Preparation of *C. parvum* oocysts antigens

*C. parvum* oocysts (P102C @ 5 × 10/6) were purchased from WaterborneTM, Inc. in the USA. The oocyst suspension, containing 2 × 10^7^ oocysts/ml of PBS, was sonicated (18,000 Hz) for twenty times at 20 s intervals for one hour (4 °C). More than 90% of the *C. parvum* oocysts were disrupted; and the undisrupted oocysts were removed by centrifuging the suspension at 1500 rpm for 15 min. The supernatant, containing oocysts antigens, was separated and divided into aliquots to be used in the immunization process.

### Production of anti-*C. parvum* oocyst antigen IgG pAbs

Five healthy and parasite free male New Zealand white rabbits (weight from 2 to 2.5 kg) were used in the immunization process for the production of pAbs against prepared *C. parvum* oocyst antigens according to the method of Tendler et al. (1991). Animals were housed under conventional laboratory settings (temperature of 21 ± 2 °C, moisture of 15%, consuming filtered water and a balanced diet containing protein (15%), fat (3%) and fibres (22%). According to Hegazy et al. (2015), Maher et al. (2022) and Farid (2023), the immunization process was accomplished through three intramuscular injections. The first priming dose (one mg of prepared *C. parvum* oocyst antigen mixed with complete Freund’s adjuvant by the ratio one to one) was followed by two boosting doses (half mg of prepared *C. parvum* oocyst antigen mixed with incomplete Freund's adjuvant by the ratio one to one) with two weeks intervals. For the purpose of determining the level of pAbs generated, blood samples were taken from the rabbit’s ear vein after four days of each boosting. Rabbits were sacrificed at the end of immunization process in order to collect blood samples. Serum was divided and stored at − 20 °C till used. Bradford method (Bradford 1976) was used to calculate the protein content of prepared antigen.

### Purification of rabbit anti-*C. parvum* oocyst antigen IgG pAbs

IgG pAb was purified by ammonium sulphate precipitation method (Nowotny 1979), caprylic acid purification method (Nowotny 1979) and diethylaminoethyl (DEAE) anion exchange chromatographic method (Mckinney and Parkinson 1987). After each step protein content of the yield was measured (Bradford 1976) in addition to sodium dodecyl-sulfate polyacrylamide gel electrophoresis (SDS-PAGE) to analyze the fractions (Thaumaturgo et al. 2002). The indirect ELISA techniques were used to evaluate the reactivity of prepared pAb (Engvall and Perlman 1971).

### Ammonium Sulfate precipitation method

In 100 ml of distilled water, 100 g of pure ammonium sulphate salt was dissolved. After two days of full dissolution, the supernatant was collected, and concentrated ammonia was added in drops to bring the pH level to 7–7.1. Rabbit serum was diluted to 50% saturation with saturated ammonium sulphate solution, continuously stirred for 20 min at 4 °C, and then centrifuged. The ammonium sulphate precipitation was again performed many times on the precipitate after the supernatant was discarded.

### Caprylic acid purification method

After the ammonium sulphate precipitation method, anti-*C. parvum* oocyst antigen IgG pAb was diluted with two volumes of sodium acetate buffer (60 mM and pH 4.8). Dropwise addition of caprylic acid (7%) was conducted for 30 min at 4 ^o^C with steady magnetic stirring. For thirty minutes, the mixture was centrifuged at 1000 rpm. Except for IgG pAb, other proteins other than albumin were precipitated. The supernatant, which contained almost pure IgG, was further refined while the precipitate was separated.

### DEAE-sephadex A-50 ion-exchange chromatography

According to their charge, proteins can often be separated via DEAE chromatography. A counter ion, such as chloride ions, can balance out the + ve charge of the DEAE group. Antibodies attach to the DEAE group less strongly than albumin because they have a more basic isoelectric point than the bulk of other serum proteins. Additionally, since it contains more lysine and arginine than glutamate and aspartate residues, IgG is more basic than IgM. Chloride ions are going to compete with the bound proteins in the binding to the + ve DEAE group, and the proteins will be eluted if the ionic strength was raised by increasing the NaCl salt content in the eluting solution. IgM and albumin are eluted later than IgG molecules. Isolated IgG fractions using this method are very pure. After being diluted in 0.5 M Tris buffer (200 ml, pH 7), the DEAE-Sephadex A-50 (Pharmacia, Uppsala, Sweden) powder was washed four times with three-bed volumes of 20 M Tris buffer (pH 7). The suspension of swollen beads was poured into a Bio-Rad column (30 × 2.5 cm) using a glass rod to prevent air bubbles. After the beads were settled in the column and the surface was covered with the binding buffer, the estimated column binding capacity was calculated. Samples were dialyzed against the binding buffer (20 mM Tris–HCl, pH 7). The column's exit tube was sealed off after the buffer over the beads was taken out. Using a Pasteur pipette, an IgG sample with the proper volume and protein content (10% of column bed capacity) was administered to the column. After the sample had penetrated the beads, the outlet tube was closed once more for 10 min to allow IgG to attach to the beads. One cc of the fraction was collected in each tube when the outlet was opened and linked to an automatic fraction collector (LKB, Dusseldoef, West Germany). Each fraction’s absorbance (280 nm) was calculated using a spectrophotometer (Perkin-Elmer Lambda 1A). High absorbance initial peak fractions were gathered together.

### Labeling of IgG pAb with horseradish peroxidase (HRP) (Periodate method)

The periodate method (Tijssen and Kurstak 1984) was used to label the prepared pAbs with HRP. After dissolving five mg of HRP in 1.2 mL of cold water, 0.3 ml of sodium periodate was added, and the mixture was allowed to sit at room temperature for 20 min. The HRP mixture was dialyzed several times against sodium acetate buffer (1 mM) at pH 4 (4 °C) overnight. The pAb IgG was diluted to 5 mg/ml and combined with 0.02 M carbonate buffer (pH 9.6). After mixing 0.5 ml of pAb solution with HRP solution, the mixture was incubated at room temperature for two hours. The solution was combined with 100 μl of sodium borohydride, which was then incubated for two hours at 4 °C. The HRP-conjugated IgG pAb was dialyzed in the presence of 0.01 M PBS at pH 7.

### Application of IgG pAbs

Six-month-old male calves were enrolled in the study. The study was conducted on 20 *C. parvum*-infected calves, 10 healthy calves; and 30 calves infected with other parasites (*Giardia, Entamoeba histolytica* and *Escherichia coli*). Blood samples (for serum preparation) and fresh fecal samples from calves were collected for examination. All samples used in the study were collected by a Veterinarian.

### Parasitological examination

Stool samples were concentrated by formalin-ether concentration method (FECT). Stool samples were carefully mixed with 10 ml of formalin (10%) before being filtered through a No. 10 mesh screen. The fecal suspension received three ml of ether before being vigorously shaken and centrifuged at 1500 rpm for 20 min. One g of the sediment was used as a fecal smear, and the supernatants were discarded. According to Farid et al. (2021), fecal smears were stained using a modified Ziehl Neelsen procedure.

### Immunological examination using IgG pAb

After standardization of the sandwich ELISA, 15 and 1/10 μg/ml were the required concentrations of IgG pAb to be used as a coating and peroxidase-conjugated antibody, respectively.

### Preparation of stool elutes

In a 25 ml polypropylene tube, each sample was combined with two parts of the dist. water and centrifuged for 15 min to create the aqueous stool elutes. To get the highest values with the least amount of background reactivity, the supernatant was serially diluted (in 2.5% FCS in PBS/T) before being tested by sandwich ELISA for antigen detection (Kamel et al. 2013).

### Detection of *C. parvum* oocysts antigens in stool and serum samples

The purified IgG pAb was applied to the plate in 100 μl/well at a concentration of 15 μg/ml, and it was then left to sit at room temperature overnight. The washing buffer (PBS/Tween) was used to wash the plate four times. Blocking buffer (2.5% FCS in PBS/T, 200 μl/well) was added to the plate, which was then incubated for two hours at 37°C. Samples (100 μl/well) were added to the wells after the plates had been washed three times followed by two hours of incubation at room temperature. HRP-conjugated pAb IgG (1/10 μg/ml) was applied to the plates. The plates were incubated at room temperature for one hour before being washed four times. O-Phenylenediamine dihydrochloride (OPD) was used as a substrate in the ELISA test. OPD was dissolved in 25 ml of phosphate citrate buffer (0.05M and pH 5), then mixed with H_2_O_2_ (5 μl). Each well received 100 μl of OPD, and the plate was incubated for 30 min at room temperature in the dark. Next, 50 μl of H_2_SO_4_ was added to each well to terminate the reaction. The ELISA plates were read at an absorbance of 492 nm.

### Application of anti-*C. parvum* oocysts IgG pAb in protection

Fifteen six-week-old healthy male balb-c mice (20–25 g) were divided in three groups (five/group); healthy uninfected control group I, *C. parvum* infected unprotected group II, *C. parvum* infected protected group III that received 0.5 ml of purified pAb with a concentration of 1/100 by subcutaneous injection. After three days of pAbs administration, mice were orally infected with 10^5^ *C. parvum* oocysts. Animals were monitored daily for symptoms of cryptosporidiosis; stool samples were collected from days 1–14 and used in detecting *C. parvum* oocysts by modified Ziehl Neelsen procedure. At the end of experiment, animals were anesthetized by an intraperitoneal injection of a combination of ketamine (30 mg/kg) and xylazine (10 mg/kg). Blood samples were collected by cardiac puncture for the preparation of serum samples (Farid et al. 2023a, b, c).

### Immunological measurements

On days 3, 7 and 14 after infection, serum level of IgA was measured by mice ELISA kits (RAB0797-1KT, sigmaaldrich, USA). Furthermore, interferon (IFN)-γ, interleukin (IL)-4 and IL-6 serum levels were measured by mice ELISA kits (ab100689, ab100710 and ab222503, respectively, Abcam, UK) according to Farid et al. (2022), Amr and Farid (2023) and Alaa et al. (2023).

### Statistical analysis

SPSS computer program (version 28) was used in the statistical analysis of the results, where, data were presented as mean (X) ± standard deviation (SD) and the cut-off value = X of healthy animals + 2(SD). The % of sensitivity, specificity, positive predictive value (PPV) and negative predictive value (NPV) were measured for each test according to Farid (2023). For results of the protection experiment (oocysts shedding, body weight and immunological results), student's t-test was used (result was considered significant when P < 0.05).

## Results

### Protein content

After each step of purification, the protein content of the yield was measured. Protein content of prepared *Cryptosporidium* oocysts antigen was 4.3 mg/ml. After the immunization period, the protein content in rabbit’s sera was 8.4 mg/ml. Finally, it became 3.2 mg/ml after the purification procedure.

### Anti-*C. parvum* oocysts IgG pAb characterization

**Fig. 1:**
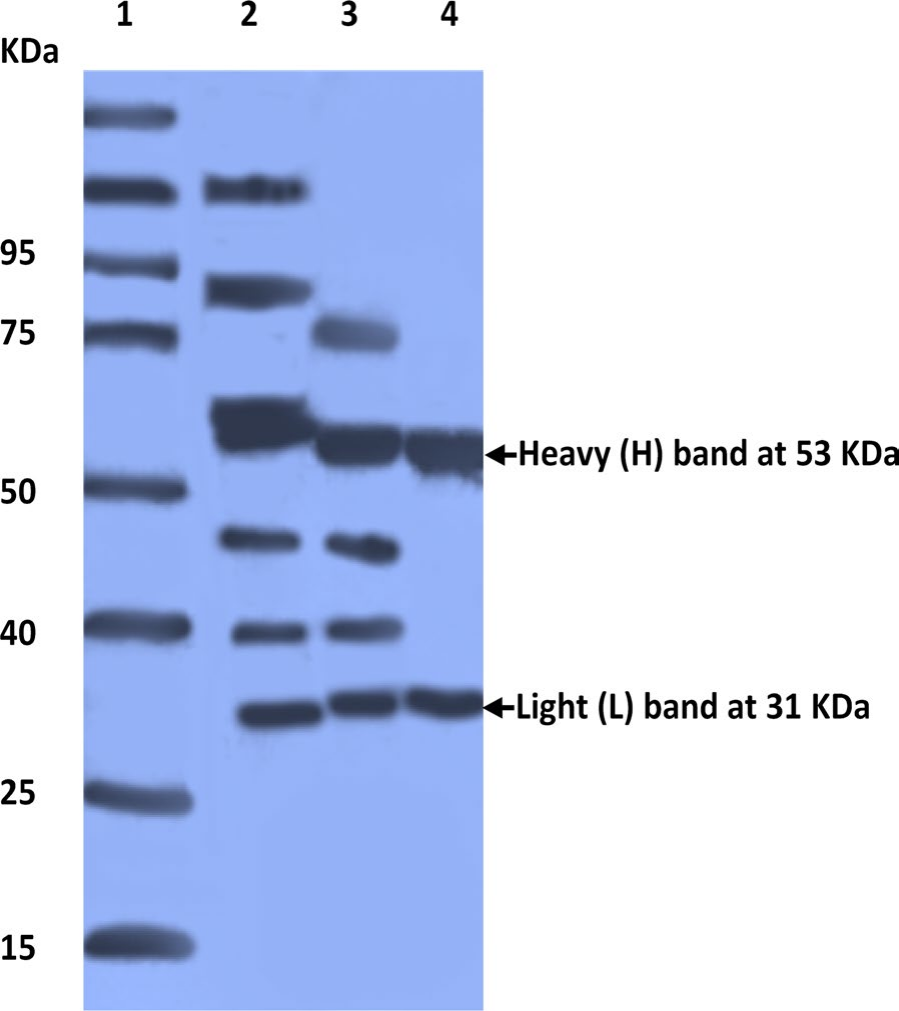
photo of 12.5% SDS-PAGE of anti-*C. parvum oocysts* IgG pAb showing molecular weight of standard protein (lane 1), proteins after 50% ammonium sulfate precipitation (lane 2), IgG pAb after 7% caprylic acid treatment (lane 3) and purified IgG pAb after ion exchange chromatography (lane 4) (coomassie blue stain)

After each step of purification, SDS-PAGE was performed to analyze the fractions and determine the purity of prepared pAbs. After all purification steps, IgG pAb was pure and free from any other proteins; and was represented by two bands (53 and 31 KDa) that represented the heavy and light chains of immunoglobulin, respectively (Fig. 1).

### Reactivity of pAbs

Indirect ELISA was used to check the reactivity of prepared pAbs against *C. parvum* oocyst antigens. The level of pAbs was elevated one week after the 1st booster dose; and 3 days after the 2nd booster dose, rabbits’ sera gave a high titer against *C. parvum* oocyst antigens at OD_492_ of 2.45.

### Parasitological results

Ordinary microscopic examination of stool samples was used to determine the validity of the results when compared to those obtained by ELISA. When stool samples were examined under microscope, 16 of the 20 calves (infected with *C.*) tested positive for *C.* oocysts, while 4 of the calves produced false-negative results. So, the assay's sensitivity was 80% and its specificity was 100%. The NPV was 90.9%, and the PPV was 100%

### Detection of *C. parvum* oocysts antigens in serum samples

The cut-off value of the test was 0.52. According to Table 1, the OD_492_ values of the *C.* infected calves (1.9 ± 0.3) were greater than those of the healthy uninfected control group (0.3 ± 0.1), as well as, those of other calves groups infected with other parasites. Three calves out of 20  *C.* infected calves produced false negative results. So, the sensitivity of the assay was 85%. The OD_492_ values of three calves out of the thirty other parasites infected calves were larger than the cut-off value producing 92.5% specificity. The PPV was 85% and the NPV was 92.5%.

**Table 1:** Detection of *C. parvum* oocysts antigen in serum samples of calves

Groups	Positive cases		Negative cases	
	Number	Mean ± SD	Number	Mean ± SD
Healthy control (n = 10)	–	–	10	0.3 ± 0.11
*C. parvum* (n = 20)	17	1.9 ± 0.32	3*	0.4 ± 0.03
*Giardia* (n = 10)	1**	0.6 ± 0.01	9	0.2 ± 0.01
*Entamoeba* (n = 10)	1**	0.6 ± 0.12	9	0.3 ± 0.01
*Escherichia coli* (n = 10)	1**	0.7 ± 0.11	9	0.4 ± 0.02

SD: standard deviation; *: false negative result; **: false positive result

### Detection of *C. parvum* oocysts antigens in stool samples

The cut-off value of the test was 0.44. One sample, only, from the *Cryptosporidium-infected* calves group gave a false negative result (Table 2) indicating 95% sensitivity and 95% PPV. One sample from *Giardia*-infected calves gave a false positive result with an OD_492_ value (0.7) higher than the cut-off value (0.44) indicating a high specificity (97.5%) and NPV (97.5%).

**Table 2:** Detection of *C. parvum* oocysts antigen in stool samples of calves

Groups	Positive cases		Negative cases	
	Number	Mean ± SD	Number	Mean ± SD
Healthy control (n = 10)	–	–	10	0.2 ± 0.12
*C. parvum* (n = 20)	19	1.7 ± 0.21	1*	0.3 ± 0.21
*Giardia* (n = 10)	1**	0.7 ± 0.31	9	0.1 ± 0.01
*Entamoeba* (n = 10)	–	–	10	0.1 ± 0.01
*Escherichia coli* (n = 10)	–	–	10	0.1 ± 0.01

SD: standard deviation; *: false negative result; **: false positive result

### Application of pAb in mice protection

**Fig. 2:**
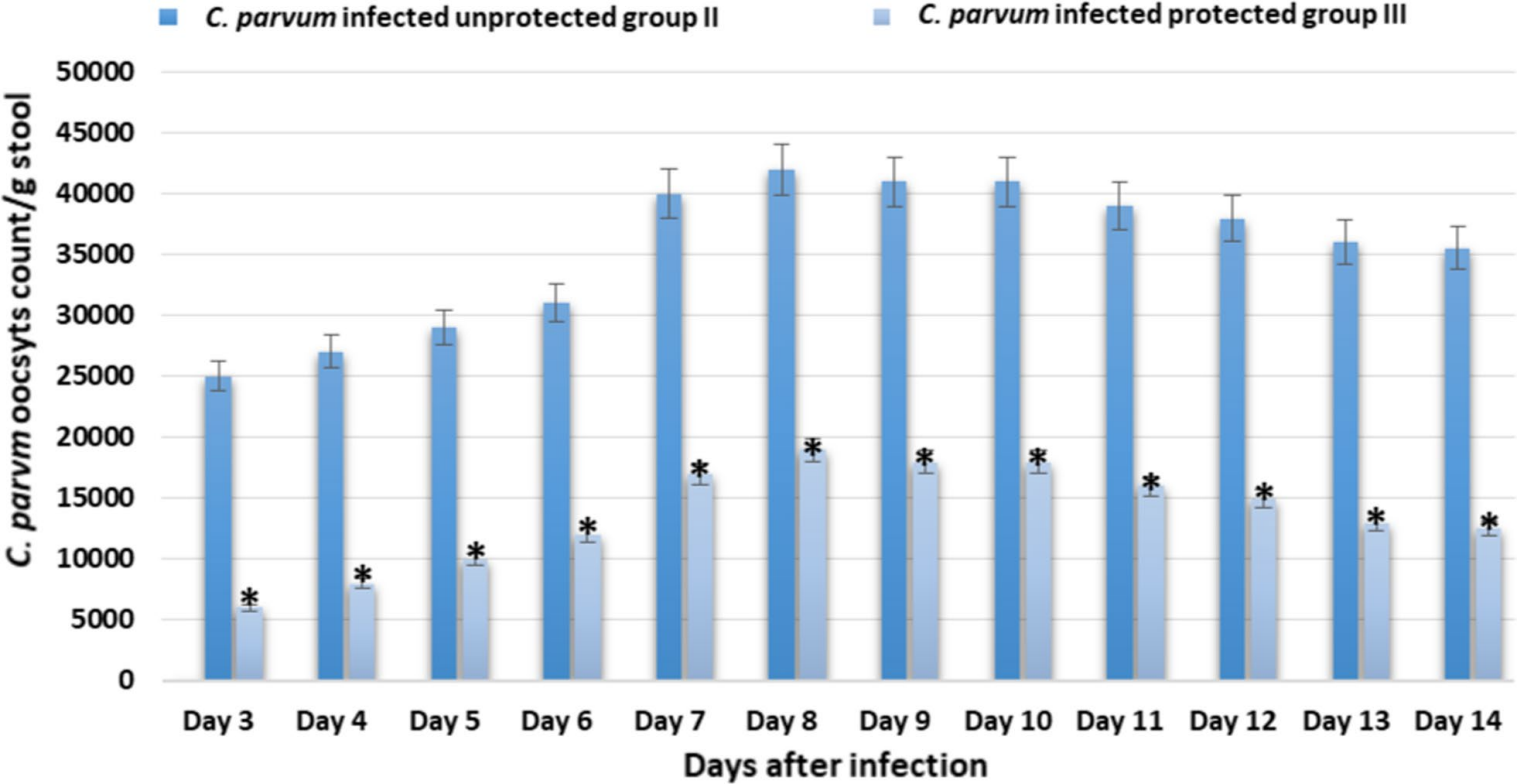
oocysts shedding from day 3 to day 14 after infection with *C. parvum*. * indicated significance (p < 0.05) in comparison to group II

**Fig. 3:**
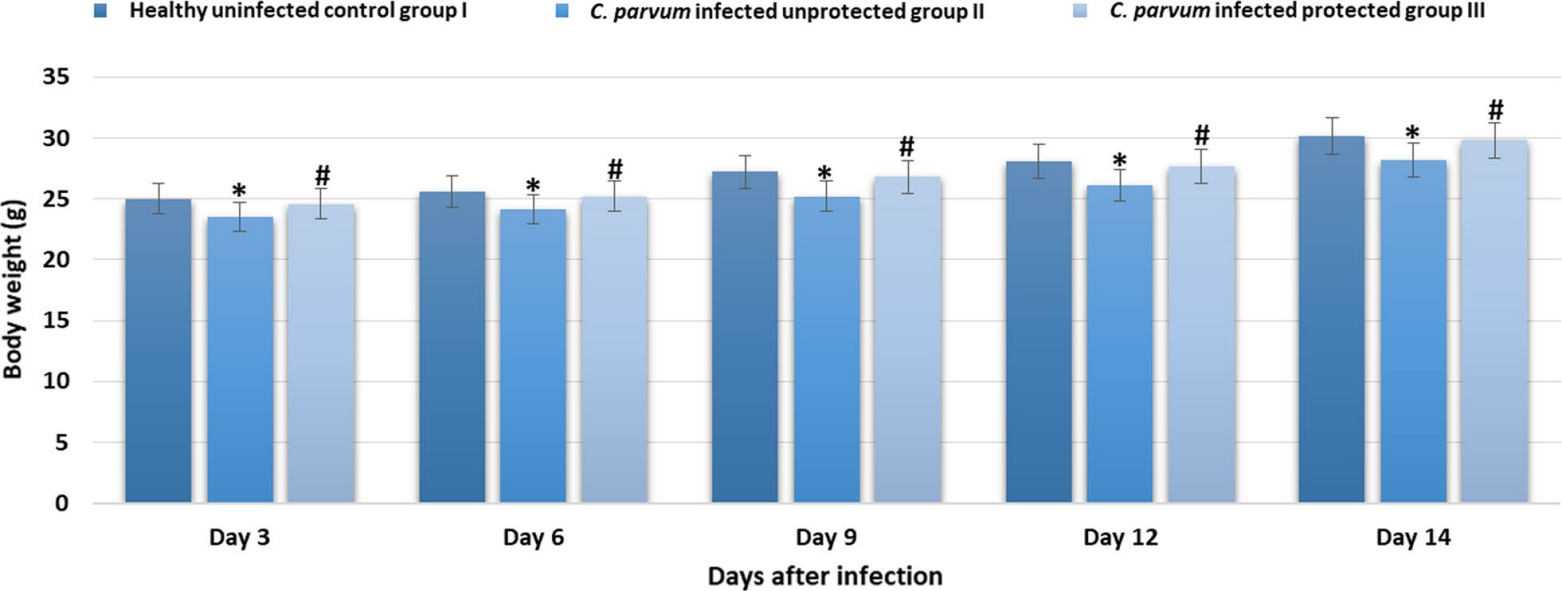
body weight of mice in different experimental groups from day 3 to day 14 after infection with *C. parvum*. * indicated significance (p < 0.05) in comparison to group I and # indicated significance (p < 0.05) in comparison to group II

Fifteen mice were divided in three groups; healthy uninfected control group I, *C. parvum* infected unprotected group II, *C. parvum* infected protected group III that received 0.5 ml of purified pAbs with a concentration of 1/100 by subcutaneous injection. After three days of pAbs administration, mice were orally infected with 10^5^  *C. parvum* oocysts and were monitored daily for symptoms of cryptosporidiosis. Infected unprotected group II showed severe diarrhea with high oocysts shedding (from day 3 to day 14) more than infected protected group III (Fig. 2). Group II was disinterested in eating and showed depression after the onset of diarrhea. Moreover, a significant reduction in the mice body weight was noticed in infected unprotected group II when compared to healthy control group I. In contrast, group III that received pAbs for protection showed no diarrhea along all experimental days and showed a non-significant reduction in body weight when compared to the healthy control group I (Fig. 3).

**Fig. 4:**
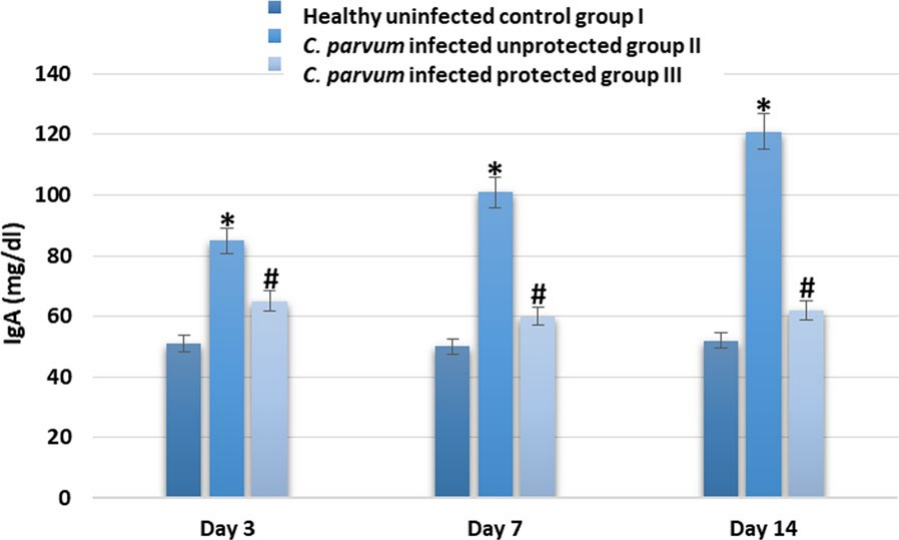
serum IgA (mg/dl) level in different experimental groups on day 3, 7 and 14 after infection with *C. parvum*. * indicated significance (p < 0.05) in comparison to group I and # indicated significance (p < 0.05) in comparison to group II

**Fig. 5:**
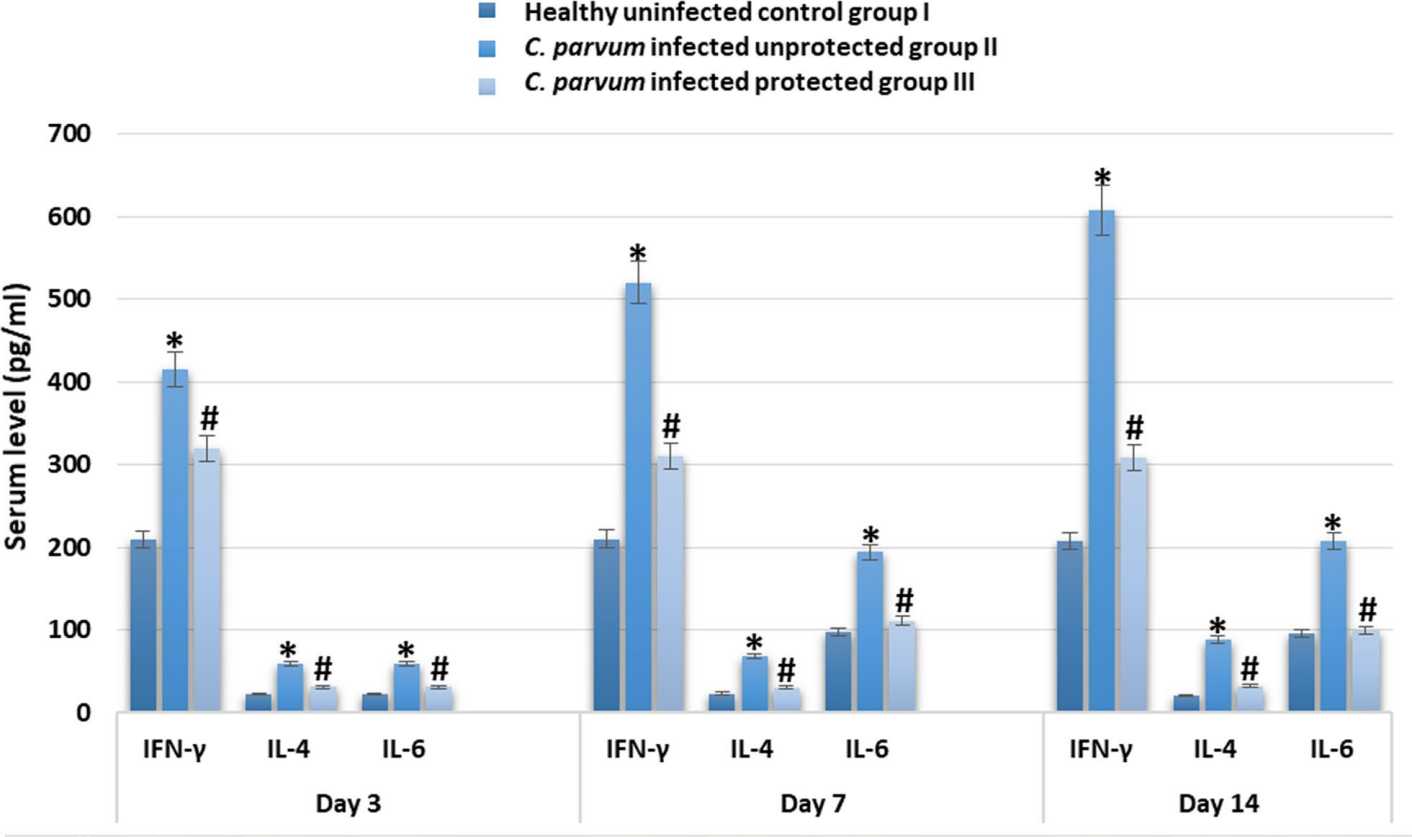
serum IFN-γ, IL-4 and IL-6 (pg/ml) level in different experimental groups on day 3, 7 and 14 after infection with *C. parvum*. * indicated significance (p < 0.05) in comparison to group I and # indicated significance (p < 0.05) in comparison to group II

Serum levels of IgA and cytokines (IFN-γ, IL-4 and IL-6) were significantly elevated in infected unprotected group II when compared to healthy control group I on days 3, 7 and 14 after infection. Levels of IgA and cytokines, in infected protected group III, were significantly lower than those of infected unprotected group II (Figs. 4 and 5).

## Discussion

Calves cryptosporidiosis is widely prevalent and has been identified as a significant contributor to calf diarrhea in several countries worldwide (Featherstone et al. 2010; Cho et al. 2014). According to Hadfield et al. (2011), either the zoonotic species *C. parvum* or the human-adapted species *C. hominis* are responsible for the majority of human cases of cryptosporidiosis. Together, these two species are responsible for 96% of the reported cases in the UK and > 90% of all human infections globally (Chalmers et al. 2011). Infection with *C.* spp., often known as cryptosporidiosis, is well acknowledged as a major cause of chronic and severe diarrhoea in immune-deficient individuals and a substantial cause of morbidity and mortality. Additionally, it is one of the significant infections responsible for zoonotic or waterborne epidemics that are mostly linked to contaminating drinking water and recreational water sites (Thompson and Ash 2016). Particularly in countries with poor infrastructure, cryptosporidiosis is recognised as a significant cause of diarrhoea in children that is linked to chronic malnutrition and early death. The dissemination of infectious oocysts causes the parasite to spread via the fecal–oral pathway. According to Painter et al. (2015), it is usually linked to contamination of food and water.

For proper care, laboratory confirmation of cryptosporidiosis is necessary, whereas when dealing with environmental specimens it is often necessary for outbreak detection, source tracing, risk factor evaluation, and intervention. Some selection standards can be used to seek for *C.* especially without the existence of a particular request, as the majority of laboratories might not test for it unless expressly requested. Currently, a number of methods may be used to diagnose *C.*, such as microscopic inspection, wet mount preparation, smears staining with modified acid-fast stain, or fluorescence stains. There are immunological techniques for detecting both antigen and antibody. Additionally accessible are several molecular techniques for DNA detection as well as histological analysis of the biopsies.

The majority of laboratories regularly employ microscopic techniques to identify cryptosporidiosis. Unfortunately, this approach has a lot of drawbacks since it needs at least 5X10^4^ oocysts per ml of faeces to be detected. Because the oocysts are shed irregularly, three samples taken on different days are thought to be optimum. Given that *C.* is a biosafety category II parasite, the sample must be handled carefully in a cabinet for safety (Khurana and Chaudhary 2018). Because of this, the parasite is difficult to be found with standard microscopy. Additionally, this method calls for advanced reporting skills. Antigen/antibody-based detection techniques like ELISA can be employed to get around all these disadvantages (Vanathy et al. 2017).

The present study aimed to prepare pAbs against *C. parvum* oocysts to be used in the diagnosis of cryptosporidiosis in serum and stool samples of infected calves. Furthermore, the prepared pAbs were tested in protection of balb-c mice from *C.* infection. The efficacy of pAbs in protection was evaluated by monitoring oocysts shedding, animals’ body weight and immunological measurements of IgA and cytokines.

For antigen preparation, *C.* oocysts were sonicated twenty times at 20 s intervals for one hour (4 °C) followed by centrifugation to obtain supernatant to be used in the immunization process. Three intramuscular shots were required to complete the immunization procedure. After the initial priming dose of one mg of prepared *C. parvum* oocyst antigen mixed with complete Freund’s adjuvant in a one-to-one ratio, two boosting doses of half mg of prepared *C. parvum* oocyst antigen mixed with incomplete Freund's adjuvant were administered at intervals of two weeks. Blood samples were obtained from the rabbit's ear vein four days after each boosting to measure the amount of pAbs produced. At the end of the immunization procedure, rabbits were sacrificed in order to collect blood for serum preparation. IgG pAb was purified by ammonium sulphate precipitation method, caprylic acid purification method and DEAE anion exchange chromatographic method. After each step protein content of the yield was measured in addition to SDS-PAGE to analyze the fractions. Indirect ELISA technique was used to evaluate the reactivity of prepared pAbs. Prepared pAbs were labelled with HRP to be used in the sandwich ELISA technique for cryptosporidiosis diagnosis in calves.

Our results showed that the protein content of prepared *Cryptosporidium* oocysts antigen was 4.3 mg/ml. After the immunization period, the protein content in rabbit’s sera was 8.4 mg/ml. Finally, it became 3.2 mg/ml after the purification procedure. After the purification steps, IgG pAb was free from any other proteins and was represented by two bands (53 and 31 KDa) that represented the heavy and light chains, respectively. The level of pAbs was elevated one week after the 1st booster dose; and 3 days after the 2nd booster dose, rabbits’ sera gave a high titer against *C. parvum* oocyst antigens at OD_492_ of 2.45. For calves’ serum and stool samples, the cut-off value of the test was 0.52 and 0.44, respectively. The sensitivity of the ELISA test was 85% and 95% for serum and stool samples; and the specificity was 92.5 and 97.5% for serum and stool samples. It was obvious that sandwich ELISA (using prepared pAbs) scored higher sensitivity than that of microscopic examination of stool samples (80%). The NPV of ELISA test (92.5% and 97.5% for serum and stool samples) was higher than that (90.9%) of the microscopic examination.

Commercially accessible kits for enzyme-labeled antibody-based ELISA for antigen detection are available. According to reports, coproantigen detection ELISA has varying detection limits of 3 × 10^5^–10^6^, which is comparable to microscopy. The sensitivity of the kit, however, may vary based on the antibody employed in some instances. Khurana et al. (2012) reported that a large number of samples can be processed quickly in the ELISA, in addition to, an excellent specificity range of 98% to 100%. Khurana and Chaudhary (2018) added that fresh, frozen, or samples that have been formalin-preserved can all be used for these assays. These benefit from quick test times and multiple findings in a single response device made the ELISA technique more superior to microscopic examination of stool samples.

Because of the oocyst stage's resilience and the absence of medications that have been licensed to prevent or treat illness, cryptosporidiosis continues to pose a serious danger to both human and animal health (Madbouly et al. 2021). It is quite common in calves, posing a risk to their health and serving as a large environmental contaminants (Riggs 2002). Passive immunotherapy using monoclonal antibodies, immune serum, or hyperimmune colostrum specific for *C. parvum* antigens has demonstrated some effectiveness against cryptosporidiosis. Although *C. parvum* oocysts continue to be shed in large quantities, passive immunotherapy using hyperimmune bovine colostrum has decreased clinical symptoms in people or dairy calves (Jenkins et al. 2004).

In addition to using pAb in diagnosis, the study evaluated the possibility of using it in protection of balb-c mice. Fifteen six-week-old healthy male balb-c mice were divided in three groups (five/group); healthy uninfected control group I, *C. parvum*-infected unprotected group II, *C. parvum* infected protected group III that received 0.5 ml of purified pAbs with a concentration of 1/100 by subcutaneous injection. After three days of pAbs administration, mice were orally infected with 10^5^ *C. parvum* oocysts. Animals were monitored daily for symptoms of cryptosporidiosis; stool samples were collected from days 1–14 and used in detecting *C. parvum* oocysts by modified Ziehl Neelsen procedure. On day 3, 7 and 14 after infection, serum levels of IgA and cytokines (IFN-γ, IL-4 and IL-6) were measured. Our results showed that infected unprotected group II showed severe diarrhea with high oocysts shedding, reduction in body weight and significant elevation in serum levels of IgA and cytokines (IFN-γ, IL-4 and IL-6) more than infected protected group III. These results indicated the usefulness of using prepared pAbs in protection against cryptosporidiosis.

To our knowledge this is the first study to use pAbs in protection against cryptosporidiosis. The prepared pAbs succeeded in the diagnosis of cryptosporidiosis in calves with high sensitivity either in serum or stool samples. The pAbs can be prepared in large amounts at low cost and used in the diagnosis and protection. We recommend the test of anti-*C. parvum* IgG pAb in protection of calves in the future. However, the study has some limitations such as the use of single concentration of pAbs (1/100) and small number of animals in the protection experiment.

## Data Availability

All data generated or analysed during this study are included in this published article.

## Abbreviations

C: *Cryptosporidium*
DEAE: Diethylaminoethyl
ELISA: Enzyme-linked immunosorbent assay
FECT: Formalin-ether concentration method
H: Heavy
HRP: Horseradish peroxidase
IFN: Interferon
Ig: Immunoglobulin
IL: Interleukin
L: Light
NPV: Negative predictive value
OPD: O-Phenylenediamine dihydrochloride
pAb: Polyclonal antibodies
PPV: Positive predictive value
SD: Standard deviation
SDS-PAGE: Sodium dodecyl-sulfate polyacrylamide gel electrophoresis
